# Competing effect of spin-orbit torque terms on perpendicular magnetization switching in structures with multiple inversion asymmetries

**DOI:** 10.1038/srep23956

**Published:** 2016-04-06

**Authors:** Guoqiang Yu, Mustafa Akyol, Pramey Upadhyaya, Xiang Li, Congli He, Yabin Fan, Mohammad Montazeri, Juan G. Alzate, Murong Lang, Kin L. Wong, Pedram Khalili Amiri, Kang L. Wang

**Affiliations:** 1 Department of Electrical Engineering, University of California, Los Angeles, California 90095, United States

## Abstract

Current-induced spin-orbit torques (SOTs) in structurally asymmetric multilayers have been used to efficiently manipulate magnetization. In a structure with vertical symmetry breaking, a damping-like SOT can deterministically switch a perpendicular magnet, provided an in-plane magnetic field is applied. Recently, it has been further demonstrated that the in-plane magnetic field can be eliminated by introducing a new type of perpendicular field-like SOT via incorporating a lateral structural asymmetry into the device. Typically, however, when a current is applied to such devices with combined vertical and lateral asymmetries, both the perpendicular field-like torque and the damping-like torque coexist, hence jointly affecting the magnetization switching behavior. Here, we study perpendicular magnetization switching driven by the combination of the perpendicular field-like and the damping-like SOTs, which exhibits deterministic switching mediated through domain wall propagation. It is demonstrated that the role of the damping-like SOT in the deterministic switching is highly dependent on the magnetization direction in the domain wall. By contrast, the perpendicular field-like SOT is solely determined by the relative orientation between the lateral structural asymmetry and the current direction, regardless of the magnetization direction in the domain wall. The experimental results further the understanding of SOTs-induced switching, with implications for spintronic devices.

Energy-efficient manipulation of magnetization through current-induced spin-orbit torques (SOTs) presents promising opportunities for applications in magnetic random access memory (MRAM) and magnetic logic devices with ultralow energy consumption, high writing speed and high endurance^1^. Fundamentally, SOTs originate from the spin-orbit coupling in structures with broken inversion symmetry, such as in nonmagnetic metal/ferromagnetic layer/insulator (NM/F/I) heterostructures, where symmetry is broken along the out-of-plane direction (*z* axis). In these structures, an in-plane current results in SOTs with both damping-like and field-like terms, due to the spin Hall^1^,^2^,^3^ and Rashba effects^4^. Experiments have already demonstrated that the damping-like SOT is capable of facilitating magnetization switching and domain wall motion in a range of structures^5^,^6^,^7^,^8^,^9^,^10^,^11^.

From low energy-dissipation, scaling and device density perspectives, switching of perpendicular magnetization driven by electric current is desirable for future generations for MRAM^12^,^13^,^14^,^15^. A damping-like SOT has been demonstrated to deterministically switch perpendicular magnetization^5^,^7^,^8^. The effective field associated with this torque can be expressed as^10^,^16^,^17^,^18^ 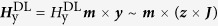 (see the coordinates in Fig. 1(b)), where ***m*** denotes the magnetization vector, ***J*** is the electrical current density vector, ***y*** is the unit vector along the *y* axis, ***z*** is the unit vector along the *z* axis, 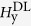 parameterizes the magnitude of the effective field per unit current density, which is determined by the material properties. In general, however, an in-plane external magnetic field (*H*_*x*_) parallel to the current flow direction (*x* axis) is required to assist the damping-like SOT to accomplish deterministic switching^5^,^8^,^16^. This is because the equilibrium magnetization state favored by the torque is in-plane^19^, and hence cannot result in a preferred perpendicular state for a given current direction. An applied *H*_*x*_ plays the role of breaking the in-plane symmetry^7^,^20^ in the structure, allowing a sole equilibrium perpendicular magnetization state for a specific current direction.

In previous works^20^,^21^, the required in-plane magnetic field was eliminated by introducing a lateral structural asymmetry in the device, which gives rise to an additional perpendicular field-like SOT. The effective magnetic field of this torque is along the *z* axis, i.e. 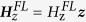. Here, the 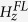 can be expressed as 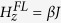, where *J* is the current density and *β* parameterizes the strength of the effective field^20^. Therefore, this effective field is able to facilitate deterministic perpendicular magnetization switching in the absence of an external field.

While bias-field-free switching of perpendicular magnetization driven by in-plane currents has been previously demonstrated in this type of structure^18^, the relative contribution of damping-like and perpendicular field-like SOTs in the magnetization switching has not yet been investigated. In this work, we study the joint effect of the perpendicular field-like and conventional damping-like SOTs in current-induced magnetization reversal by applying an in-plane bias magnetic field in addition to the lateral symmetry breaking, which in turn is realized by varying the interfacial oxidation level of the ultrathin magnetic free layer. The main difference between this work and the previous works needs to be firstly clarified. In the previous works, we have only separately studied the magnetization switching driven by the perpendicular field-like SOT in wedged samples^20^ and the conventional damping-like SOT in uniform samples^22^. In this work, we focus on studying the joint effect of the perpendicular field-like and conventional damping-like SOTs in the current-induced magnetization switching. The experimental results are consistent with a mechanism where, the perpendicular magnetization switching is determined through the competition between the perpendicular field-like SOT and the damping-like SOT. The former dominates the switching when the applied *H*_*x*_ is relatively small. On the other hand, the latter increases in importance and eventually dominates the current-driven magnetization switching as *H*_*x*_ is increased.

## Experimental Results and Discussion

**Figure 1:**
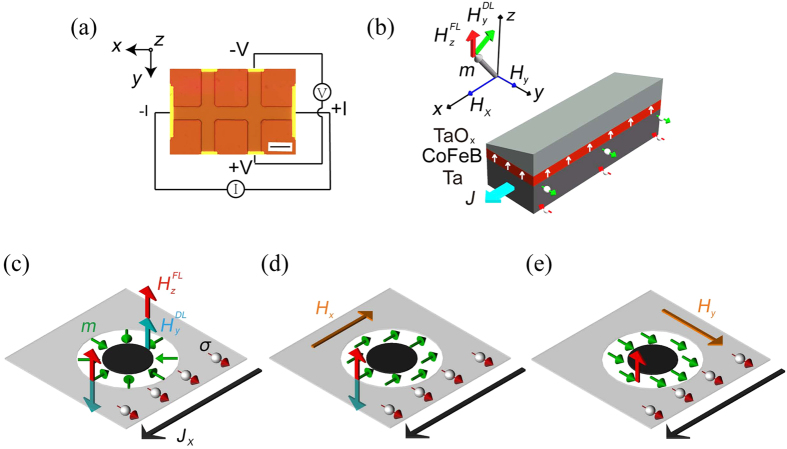
(**a**) Top view of the device consisting of a Ta/Co_20_Fe_60_B_20_/TaO_x_ (wedge) structure. The scale bar in the image is 10 *μ*m. (**b**) Schematic of the effective fields of conventional damping-like torque (Green arrow) and perpendicular field-like torque (Red arrow). The green and red arrows on the side wall of the Ta layer show the directions of the spin polarized electrons. The magnetization in the CoFeB layer is perpendicular, labeled by white arrows. (**c**,**d**) schematically show a domain, with external magnetic field *H*_*x*_ = 0 (**c**), *H*_*x*_ < 0 (**d**) and *H*_*y*_ > 0 (**e**). The gray (black) color areas show *M*_*z*_ > 0 (*M*_*z*_ < 0). The green arrows show the direction of the magnetization in the domain wall center. The red arrows with white spheres show the spin direction (σ). The black arrows show the current direction (*J*_*x*_). The directions of current-induced 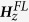 and 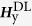 are labeled by red and light blue arrows. At *H*_*x*_ = 0, the domain wall has a right-handed chirality, which is due to the DMI. The (**d**,**e**) correspond to large enough external in-plane fields, which are able to break the DMI.

**Figure 2:**
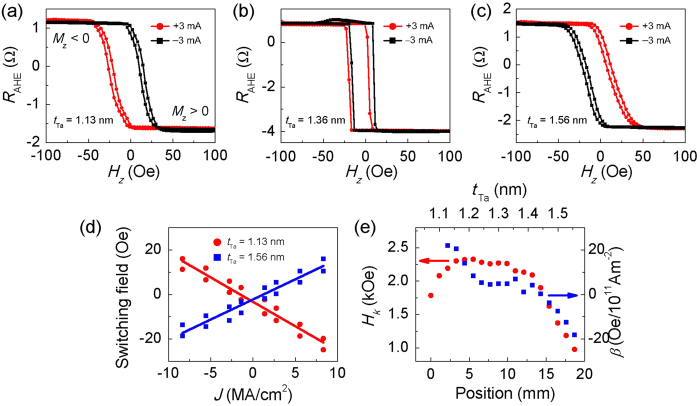
(**a**–**c**) Positive and negative current (±3 mA) induced shift of *R*_AHE_-*H*_*z*_ loops for devices A, B and C. The direction of shift induced by a positive (negative) current is negative (positive) for device A with *t*_Ta_ = 1.13 nm (before oxidation), showing that the effective field is positive (negative). The shift direction is opposite for device C with *t*_Ta_ = 1.56 nm (before oxidation). There is no obvious shift induced by current for device B with *t*_Ta_ = 1.36 nm (before oxidation). The red (black) curves correspond to positive (negative) applied current. (**d**) Current density dependence of out-of-plane switching field. The lines are linear fits to the experimental data. (**e**) Position and thickness dependence of *H*_k_ and *β*, where *H*_k_ is the in-plane saturation field and *β* parametrizes the perpendicular field-like torque term induced by the current.

Material stacks consisting of Ta(5 nm)/Co_20_Fe_60_B_20_(1 nm)/TaO_x_(wedge) were prepared on Si/SiO_2_ substrates. The TaO_x_ was prepared by oxidizing a wedged Ta layer under an O_2_/Ar plasma, where the thickness varies along the long side of substrate (*y* axis), resulting in a lateral oxidation gradient^20^. The film was annealed at 200 °C to increase the perpendicular anisotropy. After that, the films were patterned into an array of 6 *μ*m × 60 *μ*m Hall bars by standard photolithography and dry-etching techniques. The measurement configuration is shown in Fig. 1(a). The current channels of the Hall bars were along the *x* axis, as shown in Fig. 1(b). Quantitative characterizations of the effective field (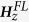) corresponding to the perpendicular field-like torque were carried out for the fabricated devices. Since the top Ta has a wedge shape, the oxidation process results in a continuous variation of oxygen content at the Co_20_Fe_60_B_20_/TaO_x_ interface along the *y* axis. This interfacial oxygen content change breaks the lateral symmetry, resulting in the perpendicular field-like torque^20^. Figure 2(a–c) show the anomalous Hall resistance (*R*_AHE_) as a function of the out-of-plane magnetic field at ±3 mA current (current density of 8.3 MA/cm^2^) for device A (*t*_Ta_ = 1.13 nm), B (*t*_Ta_ = 1.36 nm) and C (*t*_Ta_ = 1.56 nm), respectively, where *t*_Ta_ indicates the Ta thickness before oxidation. The longitudinal resistances measured by four probe method are 1.445 kΩ, 1.273 kΩ and 1.162 kΩ for three devices, respectively. As expected, the resistance decreases as the thickness of top Ta increases. For devices A and C, the current-induced 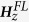 causes a shift of the AHE loops. The values of 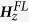 are extracted from the positive and negative switching field 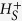 and 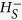, given by 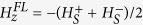. Using a linear fit, as shown in Fig. 2(d), the *β* value can be obtained from 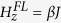, as shown in Fig. 2(e). The value of *β* is positive for thinner TaO_x_ devices. It decreases and changes its sign when the TaO_x_ thickness increases, which correlates with the change of the perpendicular anisotropy field (*H*_k_)^20^, as shown in Fig. 2(e). The opposite signs of *β* (thus the opposite 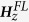) are responsible for the opposite shifts of the AHE loops in devices A and C. No obvious shift is observed in device B, indicating the current-induced 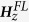 is negligible.

**Figure 3:**
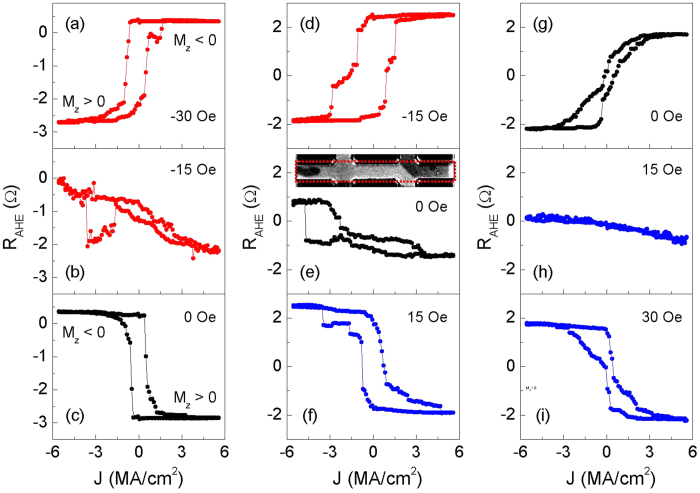
(**a**–**i**) Current induced magnetization switching at different values of *H*_x_ for devices in the region of (**a**–**c**) *β* > 0 (*t*_Ta_ = 1.15 nm), (**d**–**f**) *β* ~ 0 (*t*_Ta_ = 1.30 nm) and (**g**–**i**) *β* < 0 (*t*_Ta_ = 1.53 nm). The black curves show the case of *H*_x_ = 0 Oe. The red (blue) colors correspond to *H*_x_ < 0 Oe (*H*_x_ > 0 Oe). The inset in Fig. 3(e) shows the magnetic domain image of the 6 *μ*m × 60 *μ*m Hall bars at zero electric current, captured by the MOKE imaging experiments. The red dashed square labels the current channel. The bright (dark) areas indicate the *M*_z_ > 0 (*M*_z_ < 0).

We firstly analyze the current-induced magnetization switching driven by the damping-like SOT in the absence of perpendicular field-like SOT, i.e. in the device with *β* ~ 0. In this case, without external fields, the damping-like SOT itself cannot deterministically switch the perpendicular magnetization within the present current density range. This is evident from the measured results as shown in Fig. 3(e). The measured *R*_AHE_ values are in an intermediate state in this case even for the largest currents applied, which indicates that the device’s magnetization is in a multi-domain configuration. This is further confirmed through polar magneto-optical Kerr effect (MOKE) imaging experiments (see the MOKE image for the Hall bar device in the inset in Fig. 3(e)). When a large enough *H*_*x*_is applied, the current-induced damping-like SOT is able to drive deterministic switching of the perpendicular magnetization (see Fig. 3(d,f)). The switching is gradually accomplished as shown by the gradual variation of *R*_AHE_, indicating that the switching happens through domain nucleation and domain wall propagation, realized through domain wall depinning driven by the damping-like torque^16^. Such a switching mechanism is consistent with the previous reports on a Pt/Co/MgO structure^16^ and can be well understood by analyzing the effective field 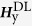 acting on the domain wall magnetization, as well as taking into account the chirality of domain walls imposed by the Dzyaloshinskii-Moriya interaction (DMI)^23^,^24^, as discussed in the following.

For a nucleated domain under zero field, as shown in Fig. 1(c), the effective field 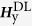 acting on the center magnetization of the domain wall is in the out-of-plane direction, which is able to drive the domain wall propagation through domain wall depinning^10^. However, the 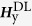 can neither shrink nor expand the nucleated domain, i.e. it cannot switch the perpendicular magnetization. This is because the current-induced 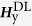 fields acting on the domain walls on the opposite sides of the domain are opposite to each other, as shown in the Fig. 1(c). This, in turn, is because the corresponding in-plane magnetizations are in opposite directions due to the domain wall chirality caused by DMI^16^. In our material system, the domain wall has a right-hand chirality (See Fig. 1(c)) as manifested in a previous experiment^22^. By applying a sufficiently large *H*_*x*_ to overcome DMI and domain wall anisotropy, the domain wall magnetizations are aligned parallel to the current, as shown in Fig. 1(d), and hence 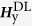 can drive switching of the perpendicular magnetization.

Next, we analyze the current-induced perpendicular magnetization switching driven by the perpendicular field-like SOT. Figure 3(c,g) show the switching for *β* > 0 and *β* < 0 devices at zero external field. For *β* > 0, a positive current produces an 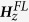 with a positive value, resulting in *M*_z_ > 0. In contrast, a positive current favors *M*_z_ < 0 for *β* < 0. Analogous to the switching for *β* ~ 0, the switching happens through domain nucleation and domain wall motion, as indicated by the gradually varying *R*_AHE_. However, in this case, the expansion of nucleated domain is accomplished by 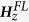 rather than by 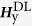, as the external field *H*_*x*_ is missing. The current-induced 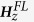
on the in-plane magnetization in the domain wall of a nucleated domain is shown in the Fig. 1(c), which is able to shrink the circular domain and favors *M*_*z*_ > 0. This signifies a key difference between 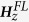 and 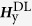, i.e. unlike 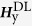, 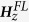 does not depend on the magnetization direction in the domain wall.

To study the joint effect of perpendicular field-like SOT and damping-like SOT in the switching, *H*_*x*_ is applied during the current-induced switching for the devices with non-zero *β* value. In this case, both of the torques play important roles in the switching. Figure 3(a) shows the current-induced switching with *H*_*x*_ = −30 Oe applied in device with *β* > 0. Positive currents favor *M*_z_ < 0, while negative currents favor *M*_z_ > 0. The favored magnetization directions are opposite compared with the switching when *H*_*x*_ = 0, as shown in Fig. 3(c). However, they are consistent with the case of the device with *β* ~ 0 when negative *H*_*x*_ is applied in Fig. 3(d), indicating the damping-like SOT dominates the switching. Figure 1(d) schematically shows the 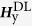 and 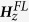 for device A with positive *β* value. As a negative *H*_*x*_ aligns the magnetization with the negative *x* axis, the 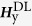 is in the –*z* direction for a positive current flow, as shown by the light blue arrows. For this direction of *H*_*x*_, 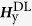 favors an opposite magnetization direction compared to 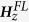. The effect of 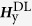 is large enough to determine the magnetization direction when *H*_*x*_ = −30 Oe is applied. When a smaller value of the in-plane field, i.e. *H*_*x*_ = −15 Oe is applied, the 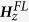 and 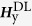 are comparable, and hence the applied current cannot completely switch the perpendicular magnetization. The decrease of 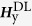 is because the alignment of in-plane magnetization in the domain wall along the *x* axis is reduced when a smaller *H*_*x*_ is applied, since the DMI favors chiral domain wall as shown in Fig. 1(c). Similar behaviors are observed in the device with *β* < 0, as shown in Fig. 3(i). When a positive *H*_*x*_ is applied, the current-induced 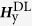 is always opposite to the 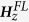. The analyses above show that, in the presence of *H*_*x*_, both 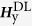 and 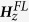 play important roles in the current-induced switching. The magnitude of *H*_*x*_ determines which SOT is predominant.

**Figure 4:**
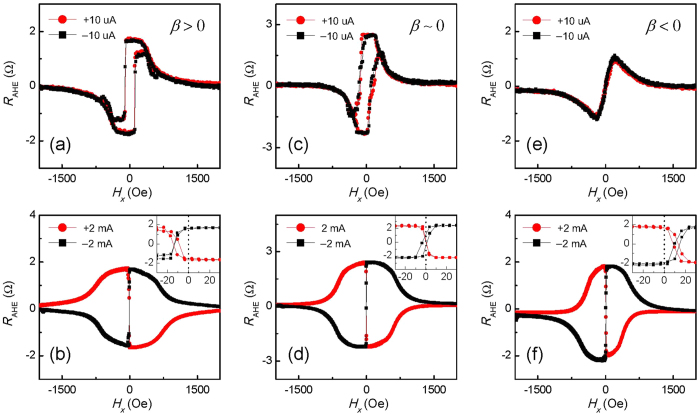
*R*_AHE_ as a function of *H*_*x*_ for devices in the region of (**a**,**b**) *β* > 0 (*t*_Ta_ = 1.18 nm), (**c**,**d**) *β* ~ 0 (*t*_Ta_ = 1.41 nm) and (**e**,**f**) *β* < 0 (*t*_Ta_ = 1.53 nm). The top three panels (**a**,**c**,**e**) show the curves at magnitude of 10 *μ*A. The bottom three panels (**b**,**d**,**f**) show the curves at current magnitude of 2 mA. The red (black) curves correspond to positive (negative) current. Insets show the data in the low magnetic field region in expanded scale.

Figure 4(a–f) show the *H*_*x*_ dependence of *R*_AHE_ for different applied currents. The observed results are consistent with those for the current-induced magnetization switching, as discussed in the following. Figure 4(a,c,e) are for current value of 10 *μ*A, which does not show obvious SOTs to affect the magnetic field-induced switching. As a result, the curves for positive and negative currents coincide with each other. When the current values are increased to 2 mA, the favored magnetization directions by positive and negative currents are opposite for relatively large *H*_*x*_ value, as shown in Fig. 4(b,d,f). The current direction determines the magnetization direction once the *H*_*x*_ value is fixed. For positive *H*_*x*_ values, the positive (negative) currents favor *M*_*z*_ > 0 (*M*_*z*_ < 0), which are consistent with results for current-driven switching. In this case, the switching at large *H*_*x*_ is dominated by current-induced 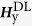. The insets in Fig. 4(b,d,f) show the low field region in expanded scale. The centers of the two loops are at *H*_*x*_ = 0 for *β* ~ 0. A current of certain direction does not favor any magnetization direction when *H*_*x*_ is zero. Consequently, the current-induced switching cannot happen, which is the case as shown in Fig. 3(e). However, the center is shifted to one side when *β* is not zero. The direction of shift depends on the sign of *β*. The results can be again interpreted by considering the 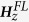 together with the 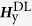, similar to the discussion in the current-driven switching experiments. For *β* > 0, the current-induced 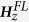 determines the favored magnetization direction at *H*_*x*_ = 0. For an *H*_*x*_ ≈ −13.2 Oe, the 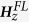 and 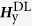 are opposite and canceled out. At larger negative *H*_*x*_ value, the 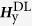 dominates the current-driven switching. The results for the *β* < 0 device can be interpreted similarly.

**Figure 5:**
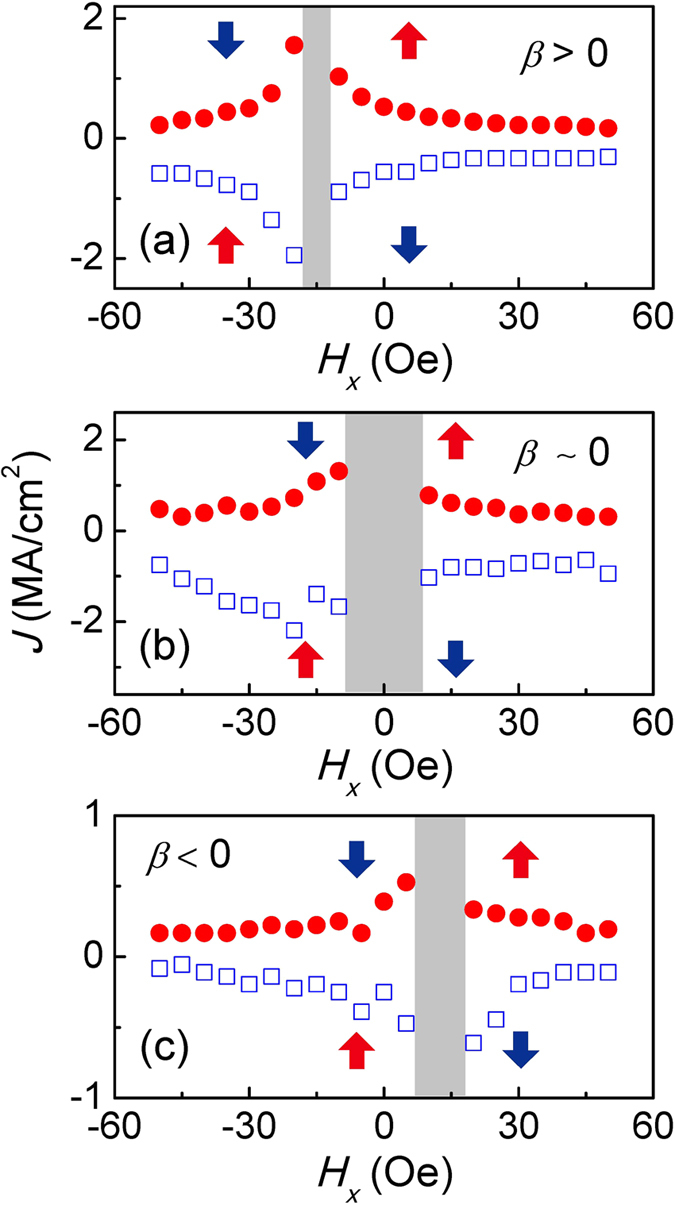
Switching phase diagram for three devices in the region of (**a**) *β* > 0 (*t*_Ta_ = 1.15 nm), (**b**) *β* ~ 0 (*t*_Ta_ = 1.30 nm) and (**c**) *β* < 0 (*t*_Ta_ = 1.53 nm), corresponding to Fig. 2, respectively. The gray color shows the area where complete switching cannot be realized within the applied current magnitude of 3 mA. The red and blue arrows show *M*_z_ > 0 and *M*_z_ < 0, respectively. The central area can allow both *M*_z_ > 0 or *M*_z_ < 0, depending on the history of applied fields and currents.

The switching phase diagram is further constructed by measuring critical switching currents at different *H*_*x*_ values. Figure 5(b) shows the switching phase diagram for device with *β* ~ 0. The phase diagram contains five different regions, where the favored magnetization directions in the four regions outside are labeled by the blue (*M*_z_ < 0) and red (*M*_z_ > 0) arrows. In the middle region, both *M*_z_ < 0 and *M*_z_ > 0 are allowed, depending on the history of magnetic field *H*_x_ and current *J*_*x*_ which are in the *x* axis. In the region labeled by gray color, current-induced switching is not allowed due to the small torques, corresponding to Fig. 3(e). As discussed above, the *H*_*x*_ is not large enough to align the domain wall magnetization along the field direction, and hence cannot produce complete switching through 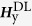 for present current range (~5.6 MA/cm^2^). This switching phase diagram is similar to previous results^7^,^16^,^22^, where the current-induced 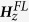 is absent. In contrast to case for *β* ~ 0, the non-reversal area is shifted to the left for device with *β* > 0 due to the current-induced 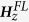. The non-reversal area shifts to right for *β* < 0. In other words, the complete switching cannot occur at specific negative (positive) *H*_*x*_ values for these two cases, as shown in Fig. 3(b,h). The mean fields (i.e. the centers of the gray area in Fig. (5)) represent the balance point where the current-induced 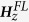 and 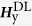 terms cancel out.

**Figure 6:**
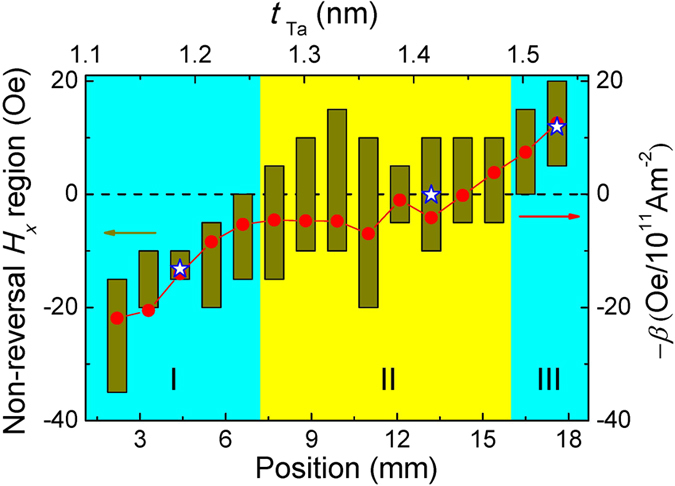
Position dependence of non-reversal field range. The non-reversal area is extracted from the switching phase diagrams, as shown in Fig. 5. The open stars show the values of the center shift obtained from the *R*_AHE_ – *H*_*x*_ loops, corresponding to the Fig. 4(b,d,f). The area II (yellow color) includes devices where complete switching cannot be achieved within the current magnitude range of 3 mA in the absence of *H*_*x*_. The areas I and III (light blue) correspond to devices where zero-field switching can be achieved.

The non-reversal *H*_*x*_ regions (vertical bars), extracted from the switching phase diagram, for the studied devices are summarized in Fig. 6, which unambiguously show the effect of 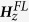 on the modification of the switching phase diagram. It is clear that the mean field values of non-reversal *H*_*x*_ regions correlate with the sign and magnitude of *β.* The reason is interpreted as follows: When *H*_*x*_ is equal to the mean field value, 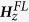 and 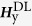 approximately cancel out, which can be expressed as 
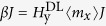
. Here, 〈*m*_*x*_〉 presents the average domain wall magnetization along the *x* axis throughout the domain walls within the device area. It is noted that the above formula is only correct when *H*_*x*_ is equal to the mean field value. For other *H*_*x*_ values in the non-reversal region, the pinning field has to be included in this expression. The DMI, by favoring a particular chirality, as shown in Fig. 1(c), tries to reduce the 〈*m*_*x*_〉 value. In contrast, the *H*_*x*_ is able to overcome DMI and domain wall anisotropy to increase the 〈*m*_*x*_〉 value. Apparently, for a larger *β* value, it requires a larger 〈*m*_*x*_〉 value to make the two torques balanced, resulting in a larger *H*_*x*_ value, i.e. the larger mean field value. On the other hand, the *H*_*x*_ value, at which 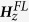 and 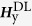 approximately cancel out, can also been extracted from the shift of *R*_AHE_ - *H*_*x*_ loop centers away from the zero field (see insets in Fig. 4(b,d,f)). The extracted values are greater and less than zero for *β* > 0 and *β* < 0, which are also shown in Fig. 6 (open stars). The values quantitatively coincide with the mean field values extracted from the switching phase diagrams. In the studied samples, the DMI value may be dependent on the TaO_x_ thickness, which is able to result in a difference between the position dependences of mean field values and *β* values. As there is not an obvious difference between these two curves, we speculate that the TaO_x_ thickness dependence of DMI magnitude may not be pronounced.

We want to point out that though the interpretation of the experimental results is based on the circular domain structures, it remains valid for other possible domain structures. In reality, the domain structures in the switching process could be very complicated and dynamically changing when a current is applied. The key of the joint effect of the two toques lies in the 〈*m*_*x*_〉 value but not the detailed domain structure. The 〈*m*_*x*_〉 value, which affects the role of 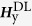, is determined by the applied in-plane magnetic field in the *x*-axis. It is also worth mentioning that the domain nucleation plays an important role in the switching, which is the first step for magnetization reversal, followed by domain wall motion. However, the switching is ultimately achieved through domain wall propagation via a depinning process driven by damping-like torque and perpendicular field-like torque, after having nucleated the domains. This is analogous to previous results also observed in structures lacking the in-plane inversion asymmetry, and hence not exhibiting the out-of-plane field-like torque^16^. The applied field *H*_*x*_ contributes to the domain wall magnetization direction in the device, and hence the domain wall propagation, thereby affecting the switching behavior. This process captures the observed dependence of the switching behavior on *H*_*x*_ in our devices. As shown in Fig. 6, the mean field values correlate very well with the *β* values, i.e. the perpendicular field-like torque, even without consideration of nucleation fields. Hence, it appears that the *H*_*x*_-dependent domain wall motion, driven by spin orbit torques captures the key experimental observations. For this reason, the details of the nucleation process are not further considered in detail in this work. In principle, a micromagnetic mechanism where an in-plane magnetic field encourages asymmetric nucleation might also contribute to the observed shifts of the non-reversal region. However, based on the present observations, we are not aware of any such mechanism in our devices. On the other hand, a description involving competition between the perpendicular field-like torque and the damping-like spin-orbit torques is shown to be consistent with the observations.

Based on Fig. 6, we can extract the regions where magnetization can be switched by current at zero field within the present current density range. In the central area (region II), the non-reversal *H*_*x*_ regions include *H*_x_ = 0. The current-induced 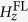 is not large enough in this case to accomplish complete deterministic switching at zero magnetic field. Alternatively, the 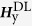 can assist the switching by applying *H*_*x*_. Within regions I and III, the non-reversal *H*_*x*_ region does not include *H*_*x*_ = 0, but rather is shifted to higher or lower in-plane field values, hence allowing for current induced switching at zero field in the present current density range. In these two regions, the required minimum *β* values for zero-field switching within the present current density range are obtained to be −5.32 × 10^11^ and 7.42 × 10^11^ Oe/Am^−2^.

As noted above, the magnitude of 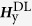 depends on the magnetization direction in the domain wall, while 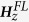 is independent of it. To further confirm this point, we also apply a transverse magnetic field (*H*_*y*_) during the current-induced switching, which tends to align the magnetization throughout the domain wall along the *y* axis, as shown in Fig. 1(e). It is expected that the 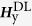 value decreases with the increase of *H*_*y*_ (reaching zero when the domain wall magnetization is along the y axis). In this case, 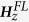 should dominate the switching, which is confirmed by experiments.

**Figure 7:**
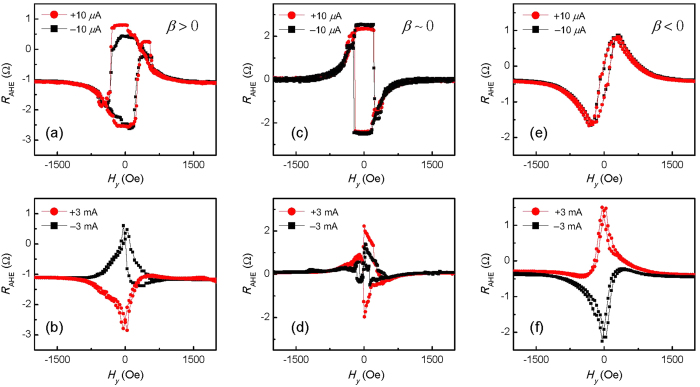
*R*_AHE_ as a function of *H*_*y*_ for three devices in the region of (**a**,**b**) *β* > 0 (*t*_Ta_ = 1.18 nm), (**c**,**d**) *β* ~ 0 (*t*_Ta_ = 1.41 nm) and (**e**,**f**) *β* < 0 (*t*_Ta_ = 1.53 nm). The top three panels (**a**,**c**,**e**) show the curves at current magnitude of 10 *μ*A. The bottom three panels (**b**,**d**,**f**) show the curves at current magnitude of 3 mA. The red (black) curves correspond to positive (negative) current.

**Figure 8:**
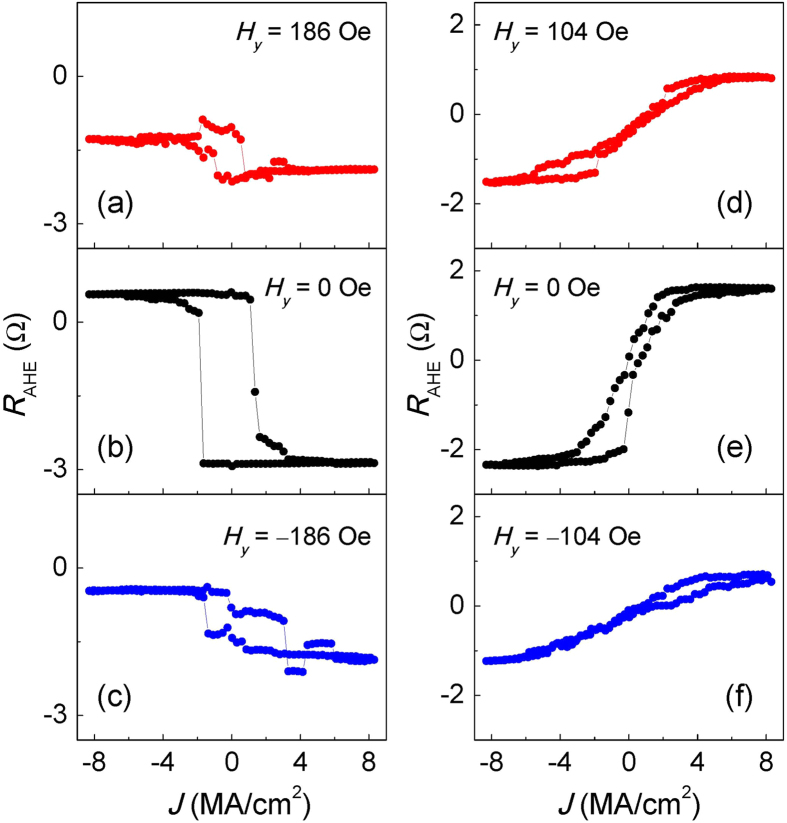
(**a**–**f**) Current-induced magnetization switching at different values of *H*_y_ for devices in the region of (**a**–**c**) *β* > 0 (*t*_Ta_ = 1.18 nm) and (**d**–**f**) *β* < 0 (*t*_Ta_ = 1.53 nm). The black curves correspond to *H*_y_ = 0 Oe. The red (blue) color shows the case of *H*_y_ < 0 Oe (*H*_y_ > 0 Oe). The 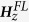 solely determines the favored perpendicular magnetization direction regardless of the direction of *H*_*y*_.

Figure 7(a) shows the *R*_AHE_ - *H*_y_ loops for *β* > 0 at ±10 *μ*A currents. The positive (negative) field favors *M*_z_ < 0 (*M*_z_ > 0), showing there is an out-of-plane component of the magnetic field due to the misalignment between the magnetic field and film plane. The two loops almost coincide with each other, confirming that the SOTs induced by current are negligible. The case is similar for *β* ~ 0 and *β* < 0, as shown in Fig. 6(c,e). When the magnitude of current is increased up to 3 mA, the effect of current appears. For *β* > 0, the positive current favors *M*_z_ > 0 in the low field range, as shown in the Fig. 7(b). This is because the 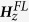 produced by positive current is along positive *z* axis, overcoming the out-of-plane component of the magnetic field. The case is opposite for *β* < 0, as shown in the Fig. 7(f). When the 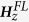 is absent, i.e. *β* ~ 0, the current of certain direction does not favor specific magnetization direction, as shown in Fig. 7(d), different to the results for *β* is not zero. The *H*_*y*_ field also tilts the *M*_z_ away from the *z* axis, giving rise to the decrease of the *R*_AHE_ at larger *H*_*y*_ values. The results demonstrate that the 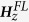 solely determines the direction of *M*_z_. This can be also seen in current-driven switching experiments. Figure 8(a–c) show the current-induced switching with a constant transverse magnetic field (*H*_*y*_) applied, *H*_*y*_ = 186 Oe, 0 Oe and −186 Oe. For all *H*_*y*_ values, positive currents favor a positive magnetization direction, which is consistent with the direction of 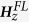. The 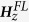 solely determines the favored perpendicular magnetization direction regardless of the direction of *H*_*y*_. Similar results are obtained for the device with *β* < 0, as shown in Fig. 8(d–f). These results further confirm that, unlike 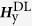, 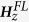 does not depend on the magnetization direction in the domain wall. On the other hand, based on symmetry arguments, *H*_*y*_ is also not expected to contribute to the switching behavior, since it does not break the symmetry of the system with respect to the *x*-*z* plane as in the case of *H*_*x*_.

## Conclusion

In summary, we studied perpendicular magnetization switching driven by conjunctional effect of the lateral asymmetry-induced perpendicular field-like SOT together with the damping-like SOT in a Ta/Co_20_Fe_60_B_20_/TaO_x_ structure with broken lateral structural symmetry. The switching was observed to occur through domain nucleation and domain wall motion. The effect of an *H*_*x*_ in deterministic switching was fully studied. When a zero or relatively small *H*_*x*_ is applied, the switching is dominated by the effect of the perpendicular field-like torque. As *H*_*x*_ is increased, the effect of damping-like SOT gradually grows and eventually dominates the magnetization switching. Current-induced switching-phase diagrams in terms of *H*_*x*_ were constructed to help visualize the corresponding effects and competition of different SOT terms. The results are relevant to the understanding and design of SOT-based magnetic memory and logic devices with perpendicular magnetization.

## Methods

The stack structure of Ta/CoFeB/TaO_x_ was fabricated from Ta(5 nm)/Co_20_Fe_60_B_20_(1 nm)/Ta(wedge) sputtered films. The metal layers were deposited on a thermally oxidized wafer (on an area of 10 mm × 50 mm) by d.c. magnetron sputtering at room temperature in an AJA International physical vapour deposition system. The top Ta was grown in a wedge shape, giving a continuous gradient of thickness along the length of the sample. The TaO_x_ layer was formed by exposing the sample to a radio-frequency O_2_/Ar plasma for 100 s. The top-wedged Ta layer was thus oxidized, resulting in a change of oxidation level at the CoFeB/TaO_x_ interface along the wedge direction. The films were then annealed at 200 °C for 30 min to enhance their perpendicular magnetic anisotropy (PMA). The magnetization of a Ta(5 nm)/Co_20_Fe_60_B_20_(1 nm)/TaO_x_ structure with uniform TaO_x_ is measured to be ~700 emu/cc. The sample was subsequently patterned into an array of Hall bar devices (seven in the width direction, with constant thickness of the top Ta layer, and thirty-five in the length direction of the sample, varying its thickness) by standard photolithography and dry etching techniques. The films were patterned into an array of 6 *μ*m × 60 *μ*m Hall bars by standard photolithography and dry-etching techniques. The Hall bar lengths were oriented along the width direction of the sample, resulting in a varying top Ta thickness (hence oxidation) along the width of the Hall bars (*i.e. y*-axis). A Keithley 6221 current source and a Keithley 2182A nano-voltmeter were used in the extraordinary Hall voltage measurement. The external magnetic field was generated by a Helmholtz coil driven by a Kepco power supply. All measurements were carried out at room temperature.

## Additional Information

**How to cite this article**: Yu, G. *et al.* Competing effect of spin-orbit torque terms on perpendicular magnetization switching in structures with multiple inversion asymmetries. *Sci. Rep.* **6**, 23956; doi: 10.1038/srep23956 (2016).
